# Author Correction: Integrative genetic analysis illuminates ALS heritability and identifies risk genes

**DOI:** 10.1038/s41467-023-43710-4

**Published:** 2023-12-04

**Authors:** Salim Megat, Natalia Mora, Jason Sanogo, Olga Roman, Alberto Catanese, Najwa Ouali Alami, Axel Freischmidt, Xhuljana Mingaj, Hortense De Calbiac, François Muratet, Sylvie Dirrig-Grosch, Stéphane Dieterle, Nick Van Bakel, Kathrin Müller, Kirsten Sieverding, Jochen Weishaupt, Peter Munch Andersen, Markus Weber, Christoph Neuwirth, Markus Margelisch, Andreas Sommacal, Kristel R. Van Eijk, Jan H. Veldink, Project Mine Als Sequencing Consortium, Géraldine Lautrette, Philippe Couratier, Agnès Camuzat, Isabelle Le Ber, Maurizio Grassano, Adriano Chio, Tobias Boeckers, Albert C. Ludolph, Francesco Roselli, Deniz Yilmazer-Hanke, Stéphanie Millecamps, Edor Kabashi, Erik Storkebaum, Chantal Sellier, Luc Dupuis

**Affiliations:** 1 Université de Strasbourg, Inserm, Mécanismes Centraux et Périphériques de la Neurodégénérescence, UMR-S1118, Centre de Recherches en Biomédecine, Strasbourg, France; 2 Department of Molecular Neurobiology, Donders Institute for Brain, Cognition and Behaviour and Faculty of Science, Radboud University, Nijmegen, Netherlands; 3 Institute of Anatomy and Cell Biology, Ulm University, Ulm, Germany; 4 German Center for Neurodegenerative Diseases (DZNE) Ulm, Ulm, Germany; 5 Clinical Neuroanatomy, Department of Neurology, Ulm University, Ulm, Germany; 6 Department of Neurology, Ulm University, Ulm, Germany; 7 Laboratory of Translational Research for Neurological Disorders, Imagine Institute, Université de Paris, INSERM UMR 1163, 75015 Paris, France; 8 Sorbonne Université, Institut du Cerveau—Paris Brain Institute—ICM, Inserm, CNRS, APHP, Hôpital de la Pitié Salpêtrière, Paris, France; 9 Institute of Human Genetics, Ulm University, Ulm, Germany; 10 Division for Neurodegenerative Diseases, Neurology Department, University Medicine Mannheim, Heidelberg University, Mannheim, Germany; 11 Department of Clinical Science, Neurosciences, Umea University, Umea, Sweden; 12 Neuromuscular Disease Unit/ALS Clinic, Kantonsspital St. Gallen, St. Gallen, Switzerland; 13 Institute for Pathology, Kanstonsspital St. Gallen, St. Gallen, Switzerland; 14 Department of Neurology, University Medical Center Utrecht Brain Center, Utrecht University, Utrecht, The Netherlands; 15 Service de Neurologie, Centre de Référence SLA et autres maladies du neurone moteur, CHU Dupuytren 1, Limoges, France; 16 ALS Center “Rita Levi Montalcini” Department of Neuroscience, University of Turin, Turin, Italy

**Keywords:** Quantitative trait, Amyotrophic lateral sclerosis, Genetics research

Correction to: *Nature Communications* 10.1038/s41467-022-35724-1, published online 20 January 2023

The original version of this article contained errors in Table 2 in the variant_location, cDNA_change, protein_change and dbSNPid columns. The correct version of Table 2 is:

**Table:** 

variant_type	variant_location	cDNA_change	protein_change	gnomAD_AF	ALS cases (*n* = 9390)	Control (*n* = 4594)	MVP	CADD	dbSNPid
missense_variant	22:g.45564104G>A	c.46G>A	p.Asp16Asn	0.000322851	11	2	0.87	26.5	rs200329756
frameshift_variant	22:g.45564091dupATAGGAATTG	c.35_45dupATAGGAATTGG	p.Asp16fs	NA	1	0	NA	NA	NA
structural	22:g.45564117A>G	c.59A>G	p.Glu20Gly	4.2e-06	3	0	0.84	26.3	rs1200142847
structural	22:g.45567544C>T	c.133C>T	p.Arg45Cys	9.68367e-05	9	0	0.89	28.1	rs113634721
missense_variant	22:g.45571835C>T	c.214C>T	p.Arg72Cys	3.23018e-05	1	0	0.81	23.6	rs781273344
splice_variant	22:g.45574119G>A	c.341G>A	p.Gly114Asp	0.000419897	3	0	0.73	24.2	rs148003438
missense_variant	22:g.45574245A>G	c.467A>G	p.Tyr156Cys	2.88367e-05	1	0	0.75	23	rs779406443
missense_variant	22:g.45574601A>G	c.823A>G	p.Lys275Glu	3.22768e-05	2	0	0.71	23.5	rs753113949
splice_acceptor	22:g.45567480G>C	c.70-1G>C	NA	3.9-06	1	0	NA	32	rs770658454
missense_variant	22:g.45574313C>G	c.535C>G	p.Pro179Ala	6.52768e-06	1	0	0.81	22.4	rs763689432
missense_variant	22:g.45580471C>T	c.1342C>T	p.Arg448Trp	6.52768e-05	1	0	0.91	24	rs777952476

which replaces the previous incorrect version:

**Table:** 

variant_type	variant_location	cDNA_change	protein_change	gnomAD_AF	ALS cases (*n* = 9390)	Control (*n* = 4594)	MVP	CADD	dbSNPid
missense_variant	22:g.45564104G>A	c.46G>A	p.Asp16Asn	0.000322851	11	2	0.88	26.5	rs200329756
frameshift_variant	22:g.45564091dupATAGGAATTG	c.35_45dupATAGGAATTGG	p.Asp16fs	NA	1	0	NA	NA	NA
structural	22:g.45564117A>G	c.59A>G	p.Gln20Cys	4.2e-06	3	0	0.86	26.3	rs1200142847
structural	22:g.45567544C>T	c.133C>T	p.Arg45Cys	9.68367e-05	9	0	0.89	28.1	rs113634721
missense_variant	22:g.45571835C>T	c.214C>T	p.Arg72Cys	3.23018e-05	1	0	0.81	23.6	rs781273344
splice_variant	22:g.45574119G>A	c.341G>A	p.Gly114Asp	0.000419897	3	0	0.74	24.2	rs148003438
missense_variant	22:g.45574245A>G	c.467A>G	p.Tyr156Cys	2.88367e-05	1	0	0.76	23	rs779406443
missense_variant	22:g.45574601A>G	c.823A>G	p.Lys275Glu	3.22768e-05	2	0	0.71	23.5	rs753113943
splice_acceptor	22:g.45567480G>C	c.70-1G>C	NA	3.9-06	1	0	NA	32	rs770658454
missense_variant	22:g.45178432C>G	c.734C>G	p.Pro179Ala	6.52768e-06	1	0	0.81	22.4	rs763689432
missense_variant	22:g.45184590C>T	c.1734C>G	p.Arg448Trp	6.52768e-05	1	0	0.91	24	rs777952476

In addition, the original version of Fig. 3c showed one variant (Lys275Glu) in an incorrect location and another variant (Arg448Trp) was missing. The correct version of Fig. 3 is:
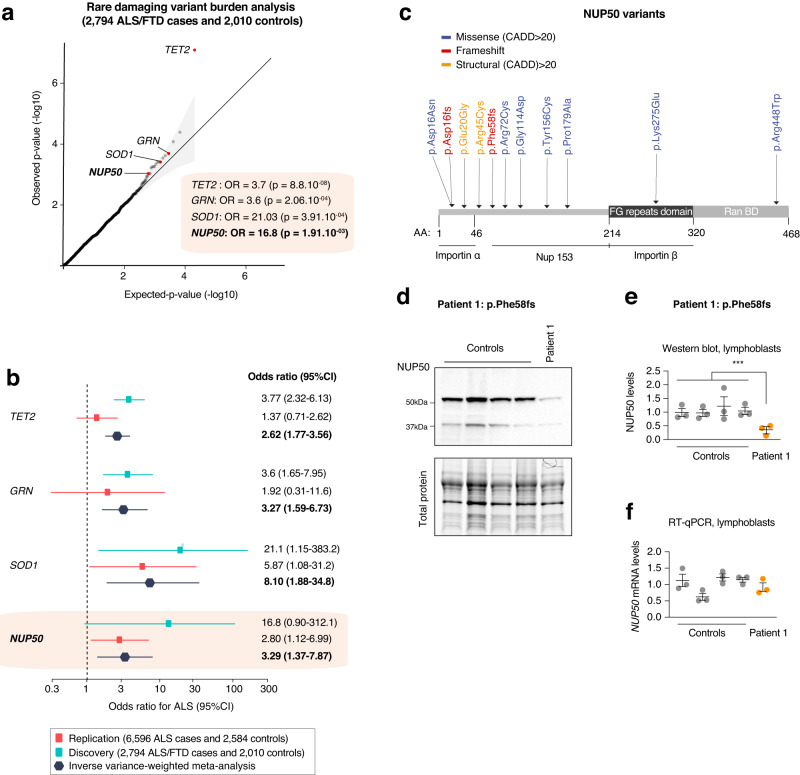


which replaces the previous incorrect version:
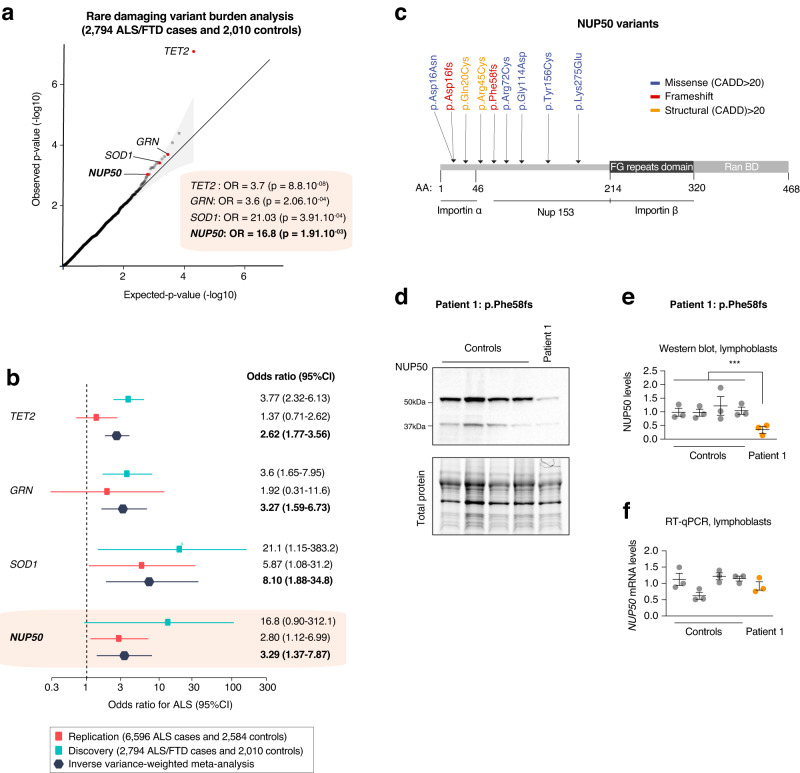


This has been corrected in both the PDF and HTML versions of the Article.

