# Supplementary material for: Integrative genetic analysis illuminates ALS heritability and identifies risk genes
Source: Nat Commun. 2023 Jan 20;14:342. doi: 10.1038/s41467-022-35724-1 (PMC9860017; doi:10.1038/s41467-022-35724-1)
Supplement: Supplementary file 1 — Supplementary Information [file 41467_2022_35724_MOESM1_ESM.pdf]

Megat et al.

Integrative genetic analysis illuminates ALS heritability and identifies novel risk genes

SUPPLEMENTARY INFORMATION

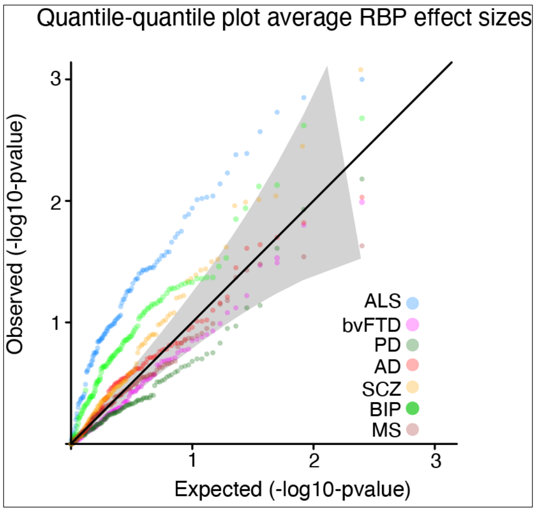

Fig. S1:Quantile-quantile plot average RBP effects binding sizes
Related to Figure 1C. Shaded gray area represent 95% confidence interval for the observed p-values.

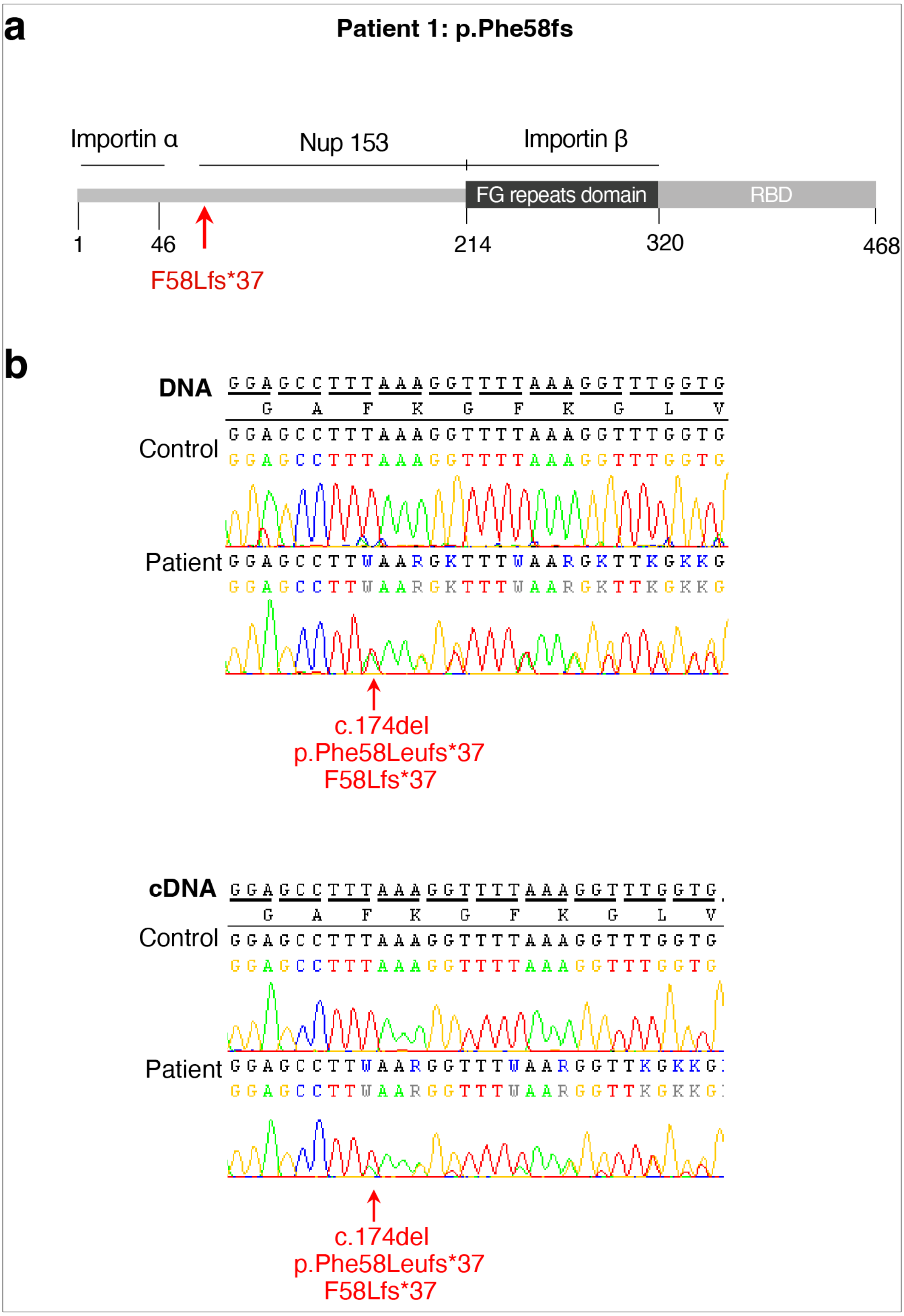

Fig. S2: *NUP50* Phe58Leufs*37 mutation

a- Representation of NUP50 protein domains with the position of the identified mutation in red. Regions of the protein interacting with importin α (Imp. α), Nup153 and importin β (Imp. β) are indicated.

b- Part of the chromatograms showing the position of the c.174del (p.F58Lfs*37) in the patient and the corresponding normal sequence (healthy control) and cDNA amplified from mRNA extracted from lymphoblasts.

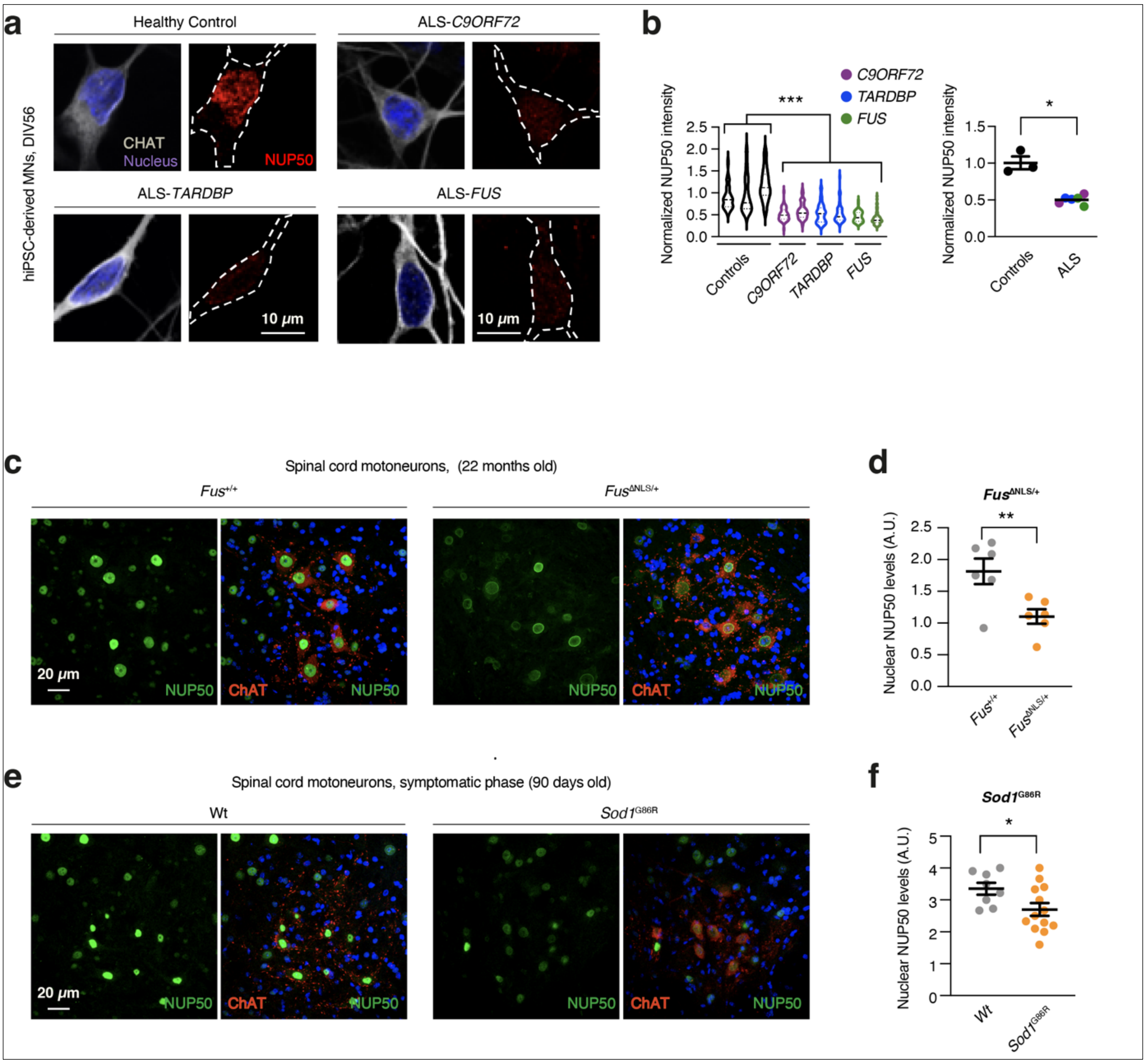

Fig. S3: NUP50 nuclear loss is a common feature in cell and animal models of ALS

a-b: Representative image of iPSCs-derived motoneurons from fALS (FUS, TDP-43 and C9ORF72 HRE carriers) immunostained for CHAT (white), nucleus (DAPI, blue) and NUP50 (red). Control 1: n= 56 cells, Control 2: n=39 cells, Control 3: n=52 cells, *C9ORF72* 1: n= 54 cells, *C9ORF72* 2: n= 53 cells, *TARDBP* 1: n=54 cells, *TARDBP* 2: n=57 cells, *FUS* 1: n= 63 cells, *FUS* 2: 65 cells, examined over 3 independent experiments. The scatter plot on the left shows the mean per subject (either control or ALS). Panel b shows quantification of NUP50 levels : Left: Kruskal Wallis, ***, p<0.001 for all comparisons between control and fALS cell lines. Right: Unpaired two tailed t-test : t=5,552, df=2,320, p = 0.0220. Data are presented as mean values +/- SEM

c-f- Representative image of immunohistochemistry for NUP50 and CHAT in spinal cord sections showing a decrease in nuclear NUP50 levels in motor neurons of (c-d) $Fus^{\Delta NLS/+}$ (Two-tailed Nested-t-test : t=3,293, df=10, p = **0,0081) and (e-f) $Sod1^{G86R}$ (Two-tailed Nested-t-test : t=2,212, df=19, p = *0.038) mice. A.U.: arbitrary units. Data are presented as mean values +/- SEM

For panel d, n=113 motor neurons examined in n=6 independent 22 months old male Wt animals, and n=132 motor neurons examined in n=6 independent $Fus^{\Delta NLS/+}$ littermates. Each dot in the scatter plot indicates the mean of an individual animal.

For panel f, n=60 motor neurons examined in n=8 independent 90 days old Wt animals, and n=106 motor neurons examined in n=13 independent $Sod1^{G86R}$ littermate animals. Each dot in the scatter plot indicates the mean of an individual animal.

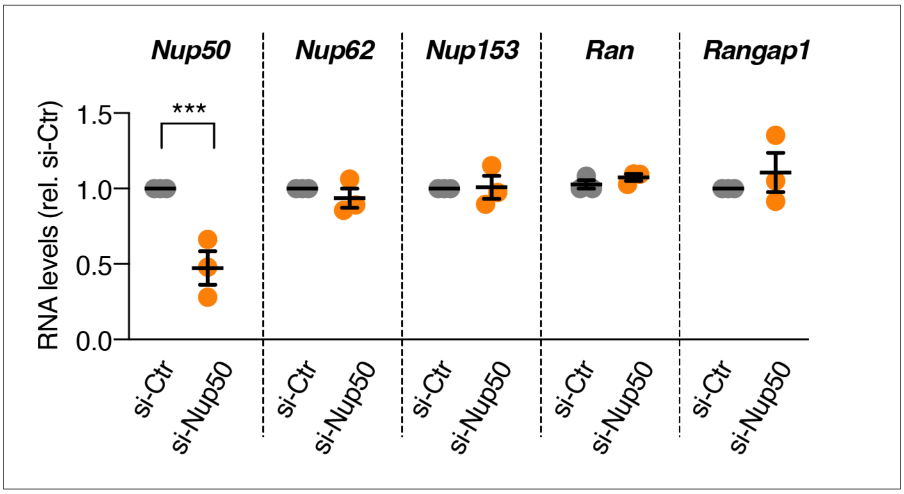

Fig. S4: mRNA levels after *Nup50* knockdown

Dot-plots showing mRNA levels of different nucleoporins after *Nup50* knock-down. Significant decrease of Nup50 expression (Two-tailed Nested t-test t=8,124, df=14, ****$p < 0.0001$) in HT22 cell lines. n=3 independent experiments performed at least in duplicate. Data are presented as mean values +/- SEM

Each dot in the scatter plot indicates the mean of an individual experiment.

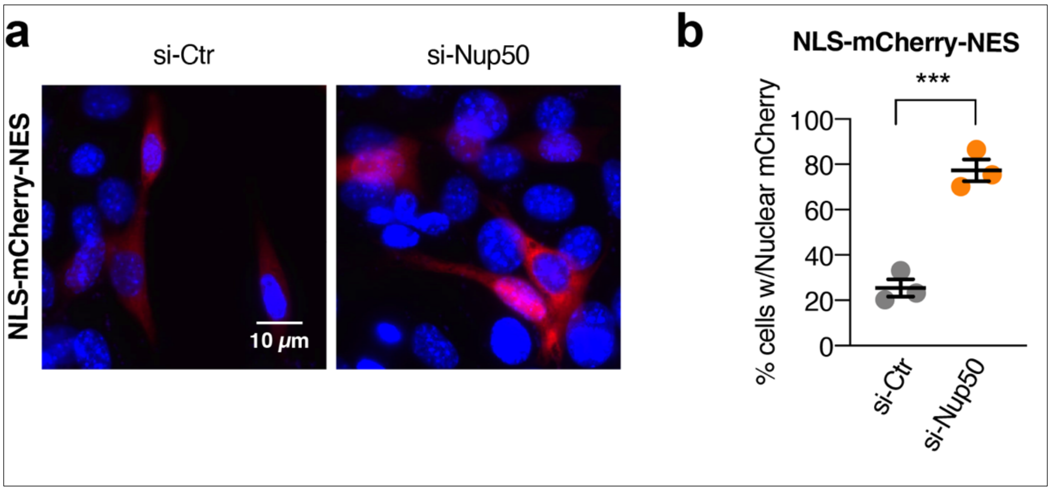

Fig. S5: *Nup50* knockdown consequences on nuclear export activity in HT22 neuronal cells.

a-b: Representative images of NLS-mCherry-NES reporter of nuclear export activity in HT22 cells transfected with si-Ctr or si-Nup50 (a), and (b) quantification (Two-tailed Nested t-test t=12,76, df=38, ****$p < 0.0001$) in HT22 cell lines. n=3 independent experiments performed at least in duplicate. Data are presented as mean values +/- SEM. Each dot in the scatter plot indicates the mean of an individual experiment.

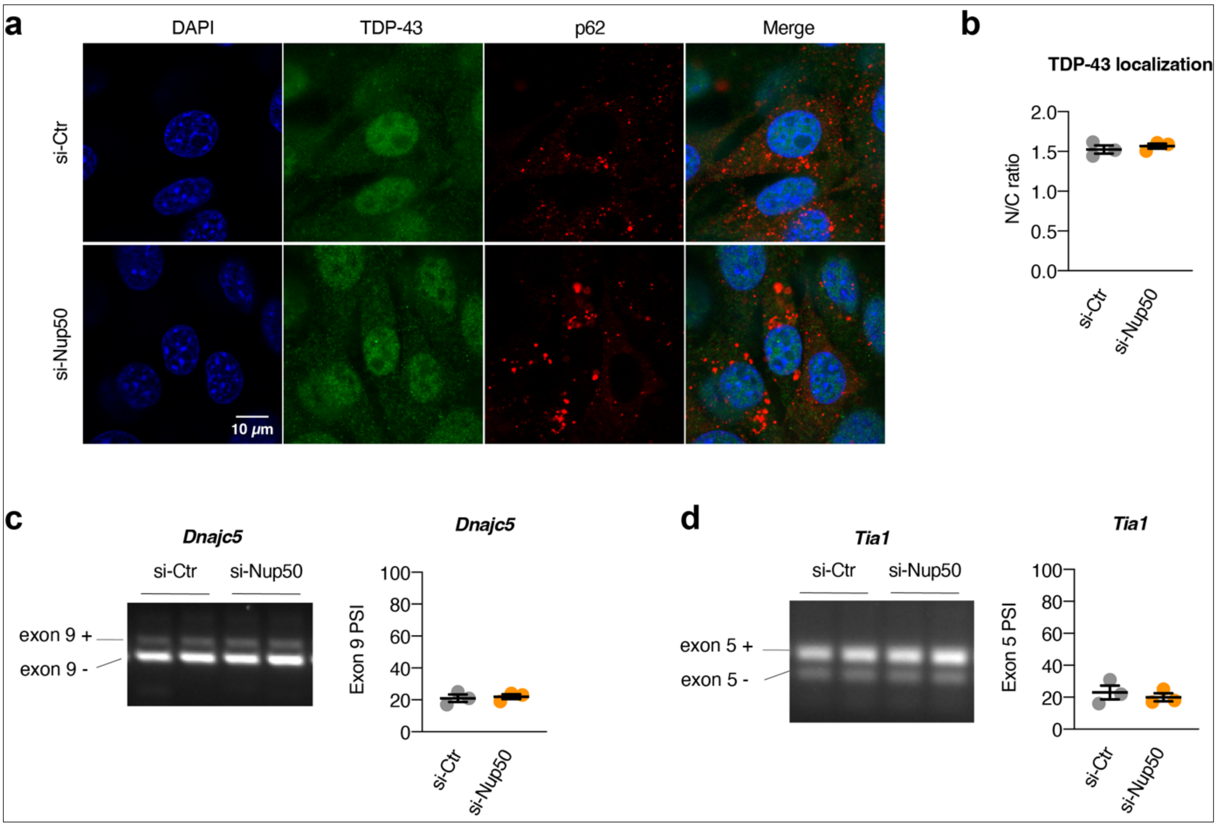

Fig. S6: TDP-43 localization and function upon *Nup50* knock-down in HT22 cells

a- representative immunofluorescence images of HT22 cells transfected either with a control siRNA (si-Ctr) or *Nup50* targeting siRNA (si-Nup50). Cells were stained 24 hours after transfection with DAPI (for nuclear staining, blue), TDP-43 (green) and p62 (red). While knockdown of *Nup50* led to p62 aggregates, we did not observe mislocalization of TDP-43. n=3 independent experiments performed at least in duplicate.

b- quantification of nuclear/cytoplasmic ratio as presented in panel a. There was no difference in TDP-43 nuclear enrichment according to *Nup50* knockdown. Data are presented as mean values +/- SEM. Data are presented as mean values +/- SEM. n=3 independent experiments performed at least in duplicate. Each dot in the scatter plot indicates the mean of an individual experiment.

c-d- splicing assays for *Dnajc5* exon 9 inclusion (C) and *Tia1* exon 5 inclusions (D), two splicing events known to be TDP-43 dependent in mouse neurons. There was no alteration in inclusion of either exon upon *Nup50* knock down. Data are presented as mean values +/- SEM. n=3 independent experiments performed at least in duplicate. Each dot in the scatter plot indicates the mean of an individual experiment.

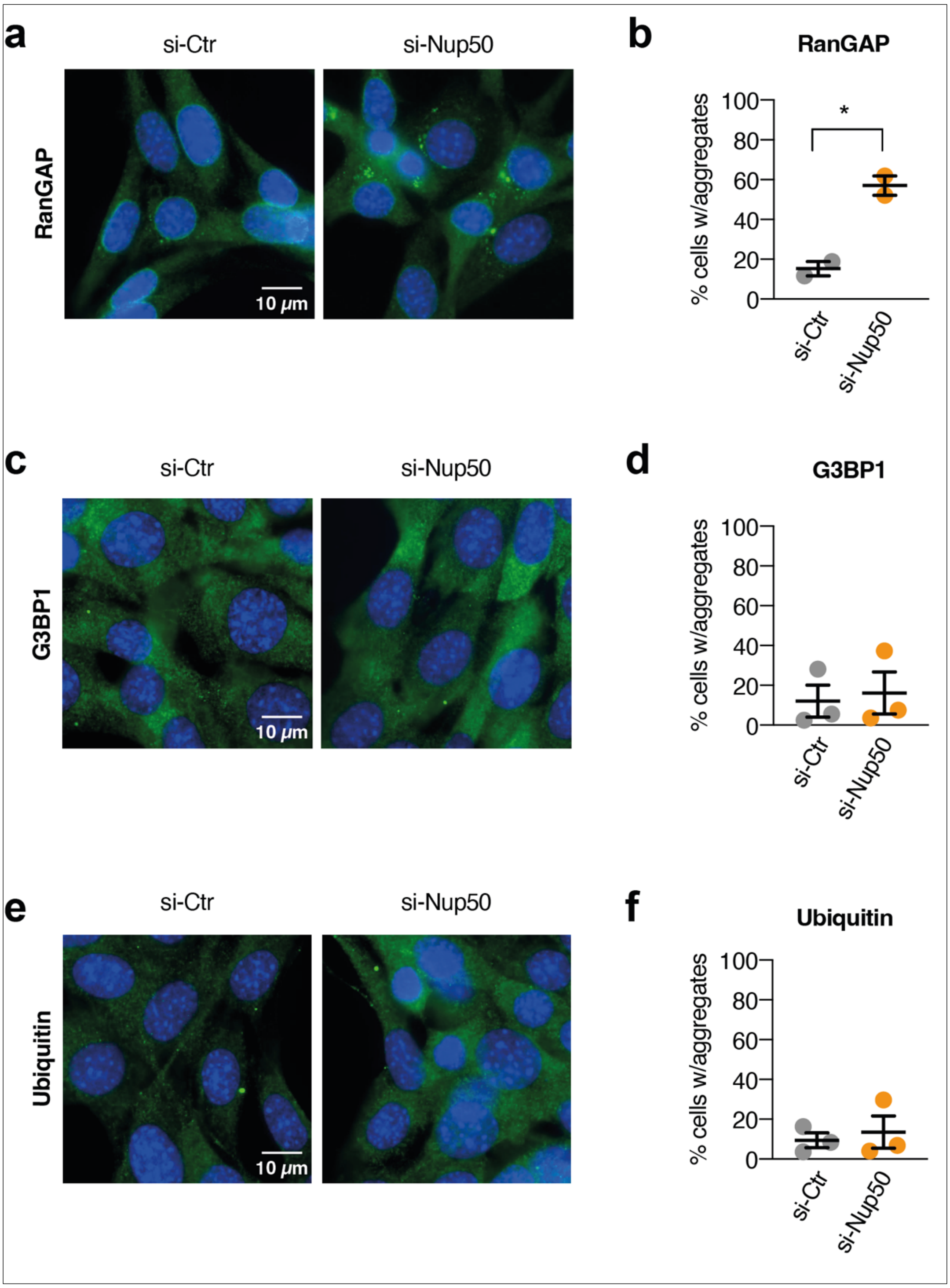

Fig. S7: additional characterization of *Nup50* knockdown in HT22 neuronal cells.
a-f- Representative images of RanGAP (a) G3BP1 (c),and Ubiquitin (e) immunofluorescence in HT22 cells transfected with si-Ctr or si-Nup50, and respective quantifications (b, d, f). Note the lack of increase in G3BP1 (Two tailed Nested t-test, t=0,6486, df=18, p=0.52) or Ubiquitin inclusions (Nested t-test , t=11.095, df=17, p= 0.28). Conversely, Nup50 knock-down increases the number of RanGAP1 inclusion (Two-tailed Nested t-test ,t=10,78, df=10, *p<0.0001). Data are presented as mean values +/- SEM. For panel a-b : n=2 independent experiments performed in triplicate. For panels c-f: n=3 independent experiments performed at least in triplicate. Each dot in the scatter plot indicates the mean of an individual experiment.

Project Mine ALS sequencing consortium members

country	name				email	affiliations
Belgium	Philip		van	Damme	philip.vandamme@uzleuven.be	KU Leuven - University of Leuven, Department of Neurosciences
France	Philippe			Corcia	corcia@med.univ-tours.fr	Centre SLA, CHRU de Tours, Tours, France; UMR 1253, iBrain, Université de Tours, Inserm, Tours, France.
France	Philippe			Couratier	philippe.couratier@unilim.fr	Centre SLA, CHRU de Tours, Tours, France.
France	Patrick			Vourc'h	vourch@med.univ-tours.fr	Service de Biochimie et Biologie moléculaire, CHU de Tours, Tours, France
Ireland	Orla			Hardiman	orla@hardiman.net	Academic Unit of Neurology, Trinity College Dublin, Trinity Biomedical Sciences Institute, Dublin, Republic of Ireland.
Ireland	Russell			McLaughlin	mclaugr@tcd.ie	Complex Trait Genomics Laboratory, Smurfit Institute of Genetics, Trinity College Dublin, Dublin, Republic of Ireland.
Israel	Marc			Gotkine	marc@gotkine.com	Department of Neurology, Hadassah Medical Organization and Faculty of Medicine, Hebrew University of Jerusalem, Israel
Israel	Vivian			Drory	vdrory@post.tau.ac.il	Department of Neurology Tel-Aviv Sourasky Medical Centre , Israel.
Italy	Nicola			Ticozzi	n.ticozzi@auxologico.it	Department of Neurology and Laboratory of Neuroscience, IRCCS Istituto Auxologico Italiano, Milano, Italy.
Italy	Vincenzo			Silani	vincenzo@silani.com	Department of Neurology and Laboratory of Neuroscience, IRCCS Istituto Auxologico Italiano, Milano, Italy.
NL	Jan	H.		Veldink	J.H.Veldink@umcutrecht.nl	Department of Neurology, UMC Utrecht Brain Center, University Medical Center Utrecht, Utrecht University, Utrecht, The Netherlands.
NL	Leonard	H.	van den	Berg	L.H.vandenBerg@umcutrecht.nl	Department of Neurology, UMC Utrecht Brain Center, University Medical Center Utrecht, Utrecht University, Utrecht, The Netherlands.
Portugal	Mamede		de	Carvalho	mamedemg@gmail.com	Instituto de Fisiologia, Instituto de Medicina Molecular,Faculdade de Medicina, Universidade de Lisboa, Lisbon, Portugal
Spain	Jesus	S.		Mora Pardina	jesussmora@icloud.com	ALS Unit, Hospital San Rafael, Madrid, Spain.
Spain	Monica			Povedano	mpovedano@bellvitgehospital.cat	la Unitat Funcional de Motoneurona, Cap de Secció de Neurofisiologia, Servei de Neurologia, Hospital Universitario de Bellvitge-IDIBELL
Sweden	Peter			Andersen	peter.andersen@umu.se	Department of Clinical Science, Neurosciences, Umeå University, Sweden.
Swiss	Markus			Weber	markus.weber@kssg.ch	Neuromuscular Diseases Unit/ALS Clinic, Kantonsspital St. Gallen, 9007, St. Gallen, Switzerland.
Turkey	Nazli	A.		Başak	nbasak@ku.edu.tr	Koç University, School of Medicine, KUTTAM-NDAL, Istanbul Turkey.
UK	Ammar			Al-Chalabi	ammar.al-chalabi@kcl.ac.uk	Maurice Wohl Clinical Neuroscience Institute, King's College London, Department of Basic and Clinical Neuroscience, London, UK.
UK	Chris			Shaw	chris.shaw@kcl.ac.uk	Maurice Wohl Clinical Neuroscience Institute, King's College London, Department of Basic and Clinical Neuroscience, London, UK.
UK	Pamela	J.		Shaw	pamela.shaw@sheffield.ac.uk	Sheffield Institute for Translational Neuroscience (SITraN), University of Sheffield, Sheffield, UK.
UK	Karen	E.		Morrison	k.morrison@qub.ac.uk	School of Medicine, Dentistry and Biomedical Sciences, Queen's University Belfast, UK.
USA	John	E.		Landers	John.Landers@umassmed.edu	Department of Neurology, University of Massachusetts Medical School, Worcester, MA, USA.
USA	Jonathan	D.		Glass	jglas03@emory.edu	Department Neurology, Emory University School of Medicine, Atlanta, GA, USA.
USA	Clifton	L.		Dalgard	clifton.dalgard@usuhs.edu	Department of Anatomy, Physiology & Genetics, Director, The American Genome Center
