# Supplementary material for: Integrative genetic analysis illuminates ALS heritability and identifies risk genes
Source: Nat Commun. 2023 Jan 20;14:342. doi: 10.1038/s41467-022-35724-1 (PMC9860017; doi:10.1038/s41467-022-35724-1)
Supplement: Supplementary file 3 — Description of Additional Supplementary Files [file 41467_2022_35724_MOESM3_ESM.pdf]

Description of Additional Supplementary Files

File Name: Supplementary Data 1
Description: Datasets used. Source of all datasets used, with hyperlinks and references.

File Name: Supplementary Data 2
Description: Oligonucleotides. Sequences of all oligonucleotides used for RT-qPCR studies in human, mouse and fly cells or tissues.

File Name: Supplementary Data 3
Description: iPSC lines used. Source and references of human iPSC lines used in Figure S3a and S3b.
