# Supplementary material for: Integrative genetic analysis illuminates ALS heritability and identifies risk genes
Source: Nat Commun. 2023 Jan 20;14:342. doi: 10.1038/s41467-022-35724-1 (PMC9860017; doi:10.1038/s41467-022-35724-1)
Supplement: Supplementary file 5 — Reporting Summary [file 41467_2022_35724_MOESM5_ESM.pdf]

nature portfolio

Corresponding author(s): DUPUIS Luc, MEGAT Salim

Last updated by author(s): Dec 2, 2022

Reporting Summary

Nature Portfolio wishes to improve the reproducibility of the work that we publish. This form provides structure for consistency and transparency in reporting. For further information on Nature Portfolio policies, see our Editorial Policies and the Editorial Policy Checklist.

Statistics

For all statistical analyses, confirm that the following items are present in the figure legend, table legend, main text, or Methods section.

n/a	Confirmed	
☐	☒	The exact sample size (*n*) for each experimental group/condition, given as a discrete number and unit of measurement
☐	☒	A statement on whether measurements were taken from distinct samples or whether the same sample was measured repeatedly
☐	☒	The statistical test(s) used AND whether they are one- or two-sided<br>*Only common tests should be described solely by name; describe more complex techniques in the Methods section.*
☐	☒	A description of all covariates tested
☐	☒	A description of any assumptions or corrections, such as tests of normality and adjustment for multiple comparisons
☐	☒	A full description of the statistical parameters including central tendency (e.g. means) or other basic estimates (e.g. regression coefficient) AND variation (e.g. standard deviation) or associated estimates of uncertainty (e.g. confidence intervals)
☐	☒	For null hypothesis testing, the test statistic (e.g. *F*, *t*, *r*) with confidence intervals, effect sizes, degrees of freedom and *P* value noted<br>*Give P values as exact values whenever suitable.*
☒	☐	For Bayesian analysis, information on the choice of priors and Markov chain Monte Carlo settings
☐	☒	For hierarchical and complex designs, identification of the appropriate level for tests and full reporting of outcomes
☐	☒	Estimates of effect sizes (e.g. Cohen's *d*, Pearson's *r*), indicating how they were calculated

Our web collection on statistics for biologists contains articles on many of the points above.

Software and code

Policy information about availability of computer code

Data collection	LasX (Leica) and Zen 2.6 softwares were used for acquisition of immunofluorescence images.
Data analysis	For genetic analyses: The following software packages were used for data analyses: R version 4.0.2 (https://www.r-project.org/), Python version 3.7 (https://anaconda.org/. PLINK version 2.0 (http://www.cog-genomics.org/plink2). ANNOVAR version 2017-07-17 for LRT, Polyphen-2, MutationTaster2, Mutation Assessor, PROVEAN and SIFT (https://annovar.openbioinformatics.org/), Polyphen-2 (http://genetics.bwh.harvard.edu/pph2/), MutationTaster2 (http://www.mutationtaster.org/), Mutation Assessor release 3 (http://mutationassessor.org/r3/). LDSC version 1.0.1 (https://github.com/bulik/ldsc), TWAS (http://gusevlab.org/projects/fusion/). SAIGE version 0.29.1 (https://github.com/weizhouUMICH/SAIGE)<br>ImageJ 1.53 software (Sun Microsystems, USA) was used for analysis of microscopy images.<br>Western blot images were analyzed using ImageLab BioRad software. qPCR were analyzed using CFX Manager software Biorad.

For manuscripts utilizing custom algorithms or software that are central to the research but not yet described in published literature, software must be made available to editors and reviewers. We strongly encourage code deposition in a community repository (e.g. GitHub). See the Nature Portfolio guidelines for submitting code & software for further information.

Data

Policy information about availability of data

All manuscripts must include a data availability statement. This statement should provide the following information, where applicable:

- Accession codes, unique identifiers, or web links for publicly available datasets
- A description of any restrictions on data availability
- For clinical datasets or third party data, please ensure that the statement adheres to our policy

The GWAS discovery/replication summary statistics and QTL annotation data generated in this study have been deposited in the zenodo database link : https://zenodo.org/record/7385500#.Y4h06uzMKBQ .

The following publicly available datasets were used in this project: NIH Genome-Wide Association Studies of Amyotrophic Lateral Sclerosis; phs000126.v1.p1, Genome-Wide Association Study of Amyotrophic Lateral Sclerosis in Finland; phs000336, Genetic Epidemiology of Refractive Error in the KORA (Kooperative Gesundheitsforschung in der Region Augsburg) Study; phs000125.v1, International Age-Related Macular Degeneration Genomics Consortium—Exome Chip Experiment; phs000187.v1. DEMENTIA-SEQ: WGS in Lewy Body Dementia and Frontotemporal Dementia; phs001963.v2.p1, AnswerALS https://www.answerals.org/
The single-cell ATACseq data used in this study are available under the accession : GSE147672
Other data generated in this study are provided in the Supplementary Information/Source Data file.

Human research participants

Policy information about studies involving human research participants and Sex and Gender in Research.

Reporting on sex and gender	Not relevant for the current study.
Population characteristics	See above
Recruitment	Description of cohorts and recruitment procedures is detailed in methods
Ethics oversight	Ethical approvals are indicated in methods and referenced manuscripts for published datasets.

Note that full information on the approval of the study protocol must also be provided in the manuscript.

Field-specific reporting

Please select the one below that is the best fit for your research. If you are not sure, read the appropriate sections before making your selection.

☒ Life sciences ☐ Behavioural & social sciences ☐ Ecological, evolutionary & environmental sciences

For a reference copy of the document with all sections, see nature.com/documents/nr-reporting-summary-flat.pdf

Life sciences study design

All studies must disclose on these points even when the disclosure is negative.

Sample size	For genetic studies, sample size was constrained by the size of the included cohorts.<br>For cell biology and animal model studies, we used samples size within the typical sample size for similar studies (see Boivin et al EMBO J. 2020 Feb 17;39(4):e100574., Picchiarelli et al, Nat Neurosci. 2019 Nov;22(11):1793-1805., Scekic-Zahirovic, Nat Commun. 2021 May 21;12(1):3028.)
Data exclusions	Data exclusion for genetics methods:<br>All multi-allelic and palindromic (A/T or C/G) SNPs were excluded. Low quality SNPs and genotyped individuals were excluded using PLINK 2.0 (--geno 0.02 and --mind 0.1). The following filter criteria were applied: MAF > 0.01, SNP genotyping rate > 0.98, Deviation from Hardy-Weinberg disequilibrium in controls P > 1 × 10-5. Then, more stringent QC thresholds were applied to exclude individuals: individual missingness > 0.02, inbreeding coefficient |F| > 0.2, mismatches between genetic and reported gender, and missing phenotypes (PLINK --mind 0.02, --het, --check-sex). Duplicate individuals were removed (king-cutoff = 0.084). Population structure was assessed by projecting 1000G principal components (PCs) and outliers from the European ancestries population were removed (> 4 SD on PC1-4).<br><br>No other data were excluded.
Replication	For TWAS: we performed a two-step TWAS in a discovery and replication cohort of patients, non overlapping.<br>For RVBA: we performed rare variant burden analysis in a discovery cohort, and the lead result was replicated in the independent Project Mine Cohort.<br>For cell biology: all experiments were performed at least 3 times, independantly. Each biological replication was performed with several

technical replicates.
For animal experiments: experiments were performed on the indicated number of animals, hence served as biological replicates.
All details on number of biological replicates are provided in the figure legends and supplementary data files.

Randomization

All our studies are based on genetic variants (both human and animal experiments). Hence randomization is not possible.

Blinding

Our human studies do not allocate to a group as we are performing genetics.
For cell biology experiments, the experimenter was blinding to treatment group for counting of immunofluorescence experiments.
In animal experiments, experimenters were blinded to the genotypes.

Reporting for specific materials, systems and methods

We require information from authors about some types of materials, experimental systems and methods used in many studies. Here, indicate whether each material, system or method listed is relevant to your study. If you are not sure if a list item applies to your research, read the appropriate section before selecting a response.

Materials & experimental systems

n/a	Involved in the study	
☐	☒	Antibodies
☐	☒	Eukaryotic cell lines
☒	☐	Palaeontology and archaeology
☐	☒	Animals and other organisms
☒	☐	Clinical data
☒	☐	Dual use research of concern

Methods

n/a	Involved in the study	
☒	☐	ChIP-seq
☒	☐	Flow cytometry
☒	☐	MRI-based neuroimaging

Antibodies

Antibodies used

For immunohistochemistry: The following antibodies were used: rabbit anti-Nup50 antibody (Ab 137092 Abcam, 1/200) and goat anti-choline acetyl transferase (AB144P, Millipore, 1/100). After 3 rinses in PBS, sections were incubated for 1h at room temperature with Hoechst (Sigma, B2261, 1/50.000) and secondary antibody: Donkey anti-rabbit Alexa-488 (JacksonImmunoResearch, 711-547-003, 1:1000) and donkey anti-goat Alexa-594 (Invitrogen, A11058, 1:1000).
For immunocytochemistry:
Cells were than incubated with the following primary antibodies: anti-CHAT (Abcam, a custom-made version of the ab181023 antibody raised in rat; diluted 1:500), anti-NUP50 (Abcam, ab137092; diluted 1:500). After overnight incubation, three washes with PBS were performed before incubating the cells with secondary antibodies (Alexa Fluor® from ThermoFisher Scientific; diluted 1:1000 in PBS) for two hours at room temperature.
For ubiquitin, RanGAP1, P62SQSTM1, Nup153, NPC, G3BP1 immunofluorescence, coverslips were incubated for 10 min in PBS with 4% paraformaldehyde, washed with PBS, and incubated in PBS plus 0.5% Triton X-100 during 10 min. The cells were washed with PBS and the coverslips were incubated during 1 h with primary antibody (1:200) against ubiquitin (3933S, Cell signaling), RanGAP1 (330800, Thermo Fisher Scientific), p62/SQSTM1 (abcam56416) Nup153 (Abcam 247000), NPC (abcam Ab24609), G3BP1 (proteintech 13057-2-AP).

Western blotting: anti-NUP50 (Abcam, ab137092, 1:1000) primary antibody

Validation

The validation of the NUP50 is shown in Figure 5a (by western blotting) eg in mouse cells: The NUP50 antibody used gave a strongly decreased signal in cells treated with NUP50 siRNA, thus indicating its specificity.
This same antibody has also been used in two other studies: Zhang K et al. Stress Granule Assembly Disrupts Nucleocytoplasmic Transport. Cell 173:958-971.e17 (2018) and Linder MI et al. Mitotic Disassembly of Nuclear Pore Complexes Involves CDK1- and PLK1-Mediated Phosphorylation of Key Interconnecting Nucleoporins. Dev Cell 43:141-156.e7 (2017).

The other antibodies were used based on their large use in multiple studies as shown on manufacturer's websites:
Chat: https://www.merckmillipore.com/FR/fr/product/Anti-Choline-Acetyltransferase-Antibody,MM_NF-AB144P (>100 reference studies)
Ubiquitin: https://www.cellsignal.com/products/primary-antibodies/ubiquitin-antibody/3933 (402 references)
RanGAP1: https://www.thermofisher.com/antibody/product/RANGAP1-Antibody-clone-19C7-Monoclonal/33-0800 (20 references)
p62: https://www.abcam.com/sqstm1--p62-antibody-2c11-bsa-and-azide-free-ab56416.html
(731 references)
Nup153: https://www.abcam.com/nup153-antibody-qe5-ab24700.html (50 references)
mAb414: https://www.abcam.com/nuclear-pore-complex-proteins-antibody-mab414-ab24609.html (138 references)
G3BP1: https://www.ptglab.com/products/G3BP1-Antibody-13057-2-AP.htm (71 references)

Eukaryotic cell lines

Policy information about cell lines and Sex and Gender in Research

Cell line source(s)	HT22 (Thermofisher)
Authentication	Cells were not authenticated
Mycoplasma contamination	cell lines were not tested for mycoplasma
Commonly misidentified lines (See ICLAC register)	No commonly misidentified cell line was used

Animals and other research organisms

Policy information about studies involving animals; ARRIVE guidelines recommended for reporting animal research, and Sex and Gender in Research

Laboratory animals	Drosophila: OK371-GAL4 (Bl 26160), UAS-mCD8-GFP (Bl 5137) and Act5C-GAL4 (Bl 4414) were obtained from the Bloomington Stock Center (Bloomington, IN). The UAS-Nup50-RNAi line was obtained from the Vienna Drosophila Resource Center (VDRC) KK library, stock number 100564.<br>Zebrafish: Danio rerio from AB strains were maintained at the Imagine Institutes (Paris) including Hb9:GFP<br>Mice: C57Bl6/J for Fus knock in mice. Fus knock in and their wild type littermate controls were 22 months old male mice. FVB/N for Sod1 G86R mice. Sod1 mice and their wild type littermate controls were 90 days old male mice.
Wild animals	The study did not involve wild animals
Reporting on sex	The information on sex was not collected in Drosophila and Zebrafish
Field-collected samples	The study did not involve samples collected from the field.
Ethics oversight	Mouse experiments were approved by local ethical committee from Strasbourg University (CREMEAS) under reference number 2016111716439395 and 25452.<br>Adult and larval zebrafish (Danio rerio) were maintained at the Imagine Institutes (Paris) fish facilities and bred according to the National and European Guidelines for Animal Welfare. Experimental procedures were approved by the National and Institutional Ethical Committees (Université de Paris). Experiments were performed on wild type and transgenic embryos from AB strains as well as Hb9:GFP zebrafish allowing the observation of motor neurons axonal arborization within a somatic segment in fixed and live animals at 48-50 hours post-fertilization larvae.

Note that full information on the approval of the study protocol must also be provided in the manuscript.
